# Tolerability and Effectiveness of Switching to Dulaglutide in Patients With Type 2 Diabetes Inadequately Controlled With Insulin Therapy

**DOI:** 10.3389/fendo.2022.880164

**Published:** 2022-06-17

**Authors:** Youngsook Kim, Ji Hye Huh, Minyoung Lee, Eun Seok Kang, Bong-Soo Cha, Byung-Wan Lee

**Affiliations:** ^1^ Division of Endocrinology and Metabolism, Department of Internal Medicine, Yonsei, University College of Medicine, Seoul, South Korea; ^2^ Division of Endocrinology and Metabolism, Department of Internal Medicine, Hallym University Sacred Heart Hospital, Anyang, South Korea

**Keywords:** GLP-1 receptor agonist, insulin therapy, type 2 diabetes, switching to GLP-1 receptor agonist, type 2 diabetes inadequately controlled with insulin therapy

## Abstract

**Aims:**

Glucagon‐like peptide 1 (GLP-1) receptor agonists have demonstrated strong glycemic control. However, few studies have investigated the effects of switching from insulin to GLP-1 receptor agonists. We aimed to investigate, using real-world data, whether switching to dulaglutide improves glycemic control in patients with type 2 diabetes mellitus (T2D) inadequately controlled with conventional insulin treatment.

**Materials and methods:**

We retrospectively evaluated 138 patients with T2D who were switched from insulin to dulaglutide therapy. We excluded 20 patients who dropped out during the follow-up period. The participants were divided into two groups according to whether they resumed insulin treatment at 6 months after switching to a GLP-1 receptor agonist (group I) or not (group II). A multiple logistic regression analysis was performed to evaluate the parameters associated with the risk of resuming insulin after replacement with dulaglutide.

**Results:**

Of 118 patients initiated on the GLP-1 receptor agonist, 62 (53%) resumed insulin treatment (group I), and 53 (47%) continued with GLP-1 receptor agonists or switched to oral anti-hypoglycemic agents (group II). Older age, a higher insulin dose, and lower postprandial glucose levels while switching to the GLP-1 receptor agonist were associated with failure to switch to the GLP-1 receptor agonist from insulin.

**Conclusions:**

A considerable proportion of patients with T2D inadequately controlled with insulin treatment successfully switched to the GLP-1 receptor agonist. Younger age, a lower dose of insulin, and a higher baseline postprandial glucose level may be significant predictors of successful switching from insulin to GLP-1 receptor agonist therapy.

## Introduction

For patients with type 2 diabetes (T2D) having uncontrolled glucose levels, insulin therapy has been traditionally considered as the most effective treatment available for managing hyperglycemia, and, more commonly, as an adjunct to oral hypoglycemic agents (OHAs) (1–3). However, approximately 40% of patients treated with insulin ultimately fail to achieve their target HbA1c levels and require insulin intensification (4). Although insulin intensification is theoretically the best treatment option for glycemic control (5, 6), in clinical practice, it does not successfully maintain glycemic control. Moreover, insulin intensification, such as that by a multiple daily injection regimen, frequently increases the risks of adverse events such as weight gain and hypoglycemia (7, 8). Consequently, it may lead to overall dissatisfaction with the treatment and poor compliance with therapy in patients with T2D (8) Moreover, HbA1c goals are often unmet even after increasing the number and dose of insulin injections in real practice.

As per recent research, the intestine, brain, kidney, and immune system are emerging targets for the treatment of diabetes (9); therefore, glucagon-like peptide-1 (GLP-1) receptor agonists that target pancreatic beta and alpha cells, the intestine, and the brain have been developed and are widely used to regulate glucose metabolism (10). Several recent studies have demonstrated that GLP-1 receptor agonists are as effective as insulin regimens in lowering HbA1c (11, 12).

However, few studies have been conducted on whether patients with uncontrolled T2D could be successfully switched from insulin therapy to GLP-1 receptor agonist therapy. Moreover, there is limited data on the clinical characteristics that predict the successful continuation of GLP-1 receptor agonists after switching from an insulin regimen. Therefore, the present study aimed to investigate whether switching to dulaglutide, a weekly injectable GLP-1 receptor agonist, from insulin improves glycemic control in patients with T2D inadequately controlled with conventional insulin treatment.

## Materials and Methods

### Study Design and Data Source

**Figure 1:**
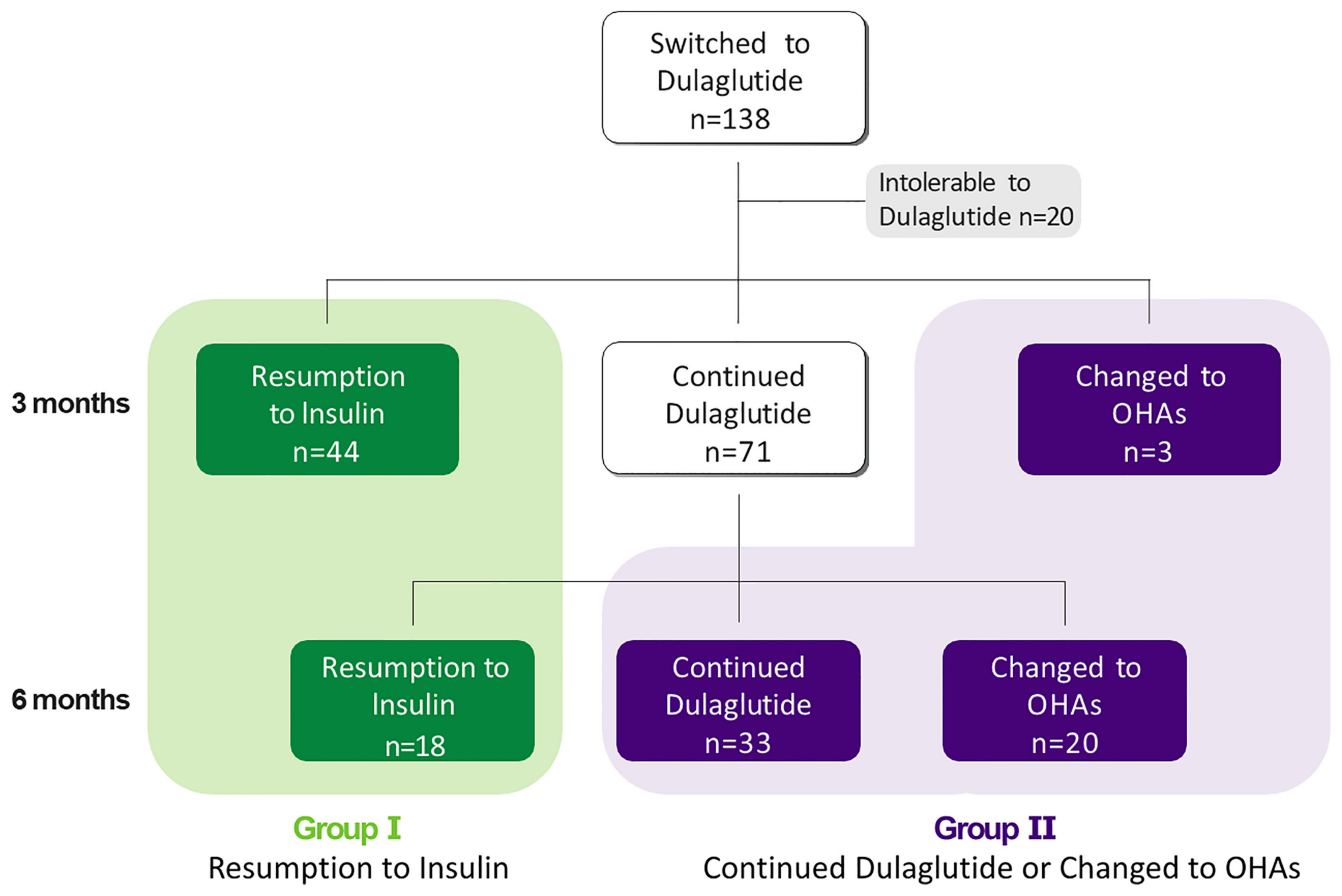
A total of 138 patients switched from insulin to dulaglutide. 20 patients were not resistant to Dulaglutide and 118 patients were followed for six months after conversion. 44 patients resumed insulin, 71 patients continued to use dulaglutide. And three patients switched from dulaglutide to oral hypoglycemic agents after three months. Additional 18 patients resumed insulin, 33 patients continued using dulaglutide and 20 patients switched to OHA after six months.

In this retrospective, observational study, we analyzed the human subjects’ medical record and laboratory data of 138 patients with T2D whose HbA1c levels were 7.6% or higher when treatment was switched from insulin to dulaglutide with OHAs between July 2017 and March 2021. Although this study is retrospective, the researcher’s own supervision and the Institutional Review Board’s deliberation were conducted on the data processing. The protocol of this study adhered to the tenets of the Declaration of Helsinki and Korean Good Clinical Practice and was approved by the Institutional Review Board (IRB No. 4-2021-1639) of Severance Hospital. The requirement of written informed consent was waived because the data were accessed only for analysis, and personal information was not used. We reviewed the electronic medical records to assess whether the subjects who stopped using dulaglutide later resumed insulin therapy or switched to OHAs after discontinuing dulaglutide over the 6 months. For the effectiveness of analysis, 20 patients who dropped out for various reasons were excluded, and the remaining study subjects were first classified into two groups according to the resumption of insulin therapy during the 6 months: the resumption-to-insulin group (group I, n= 62) and the continued-dulaglutide-or-changed-to-OHAs group (group II, n=56) (**Figure 1**). Group I was further divided into group Ia (n=44), in which insulin was replaced before or at the first visit, and group Ib (n=18), in which insulin was replaced six months after switching to dulaglutide.

Because this was a retrospective and real-world data study, the decisions of switching to and continuing with dulaglutide, switching to only OHAs, or resuming insulin were fully at the discretions of the physicians based on their clinical judgments and the patients’ blood glucose parameters. However, OHAs were usually given in combination with metformin and sulfonylurea, according to the health insurance policy, and there were rare exceptions.

### Clinical and Laboratory Measurements

The baseline demographic and clinical characteristics of the patients were included. Body mass index (BMI) was defined as body weight divided by the square of the height in meters (kg/m^2^). Hypertension and dyslipidemia were confirmed by diagnoses and prescription medications present in the medical records.

The baseline laboratory parameters were also included. Fasting and postprandial glucose levels were measured using the hexokinase method, and enzyme colorimetry was used to measure total cholesterol, triglyceride, and high-density lipoprotein cholesterol levels. The HbA1c level was determined by high-performance liquid chromatography using Variant™ II Turbo (Bio-Rad Laboratories, Her-clusters, CA, USA). Serum C-peptide levels were measured in duplicates using immunoradiography (Beckman Coulter, Fullerton, CA, USA). Glycated hemoglobin, fasting glucose, and postprandial glucose levels were checked at the first and second visits to assess the glycemic efficacy after switching from insulin to dulaglutide therapy.

### Statistical Analysis

Continuous variables are expressed as mean ± standard deviation, and categorical variables are expressed as percentages. Data are presented as the mean ± standard error (SE). Differences between the two groups were analyzed using Student’s t-test for continuous variables and chi-square test for categorical variables. A logistic regression analysis was performed to assess whether continuing with dulaglutide/changing to OHAs or resumption of insulin treatment after the switch to dulaglutide were associated with clinical and laboratory parameters at the baseline. The receiver operating characteristic curve of the total insulin dose was used to determine the optimal cut-off value for the prediction of successful continuation of dulaglutide therapy by using the area under the curve with a maximum Youden index (sensitivity+specificity-1). Statistical significance was set at p<0.05. Statistical analyses were performed using PASW Statistics version 26.0 for Windows (SPSS Inc., Chicago, IL, USA).

## Results

### Patient Flow and Classification

Of the 293 patients with T2D who switched to weekly dulaglutide from insulin, 138 patients were finally enrolled in this study. During the follow-up period, 20 patients stopped using dulaglutide for various reasons, such as gastrointestinal disturbances and patient preferences. Approximately 86% of the enrolled subjects tolerated weekly dulaglutide treatment. Thereafter, 118 subjects were included for the effectiveness and predictive factor analyses. Among them, 62 (53%) resumed insulin by the end of the follow-up period (group I). Of them, 44 patients resumed insulin at the first visit at 3 months and 18 patients resumed insulin at the second visit at 6 months. Fifty-six (47%) patients continued with dulaglutide or switched to OHAs without restarting insulin (group II) (**Figure 1**). All patients started with dulaglutide at 0.75 mg for initial 1 month and then increased to 1.5 mg if tolerated. 96.6% of patients included in the study maintained 1.5 mg of dulaglutide during the maintenance period, except for the first adaptation period.

### Baseline Clinical and Laboratory Characteristics of the Patients

The baseline clinical and laboratory characteristics of the patients in the effectiveness analysis set are shown in **Table 1**. The presence of glutamic acid decarboxylase (GAD) antibody was not found in enrolled subjects. The mean age and the mean duration of T2D were 60.4 ± 11.7 and 14.2 years, respectively. The proportion of male were 59%, 63% and 55% in all enrolled patients, group 1, and group 2, respectively. The average BMI was 27.42 ± 3.50 kg/m^2^. The duration of insulin use was > 6 months in all the patients. HbA1c and fasting and postprandial glucose were measured in all patients at every visit. We adopted HbA1c and fasting and postprandial glucose as patient’s blood sugar levels in this study. The baseline HbA1c level (group I vs. group II, 8.56 ± 0.78% vs. 8.69 ± 1.08%, p= 0.802), the fasting (140.8 ± 50.2 mg/dL vs. 148.1 ± 53.8 mg/dL, p=0.351) and postprandial (213.5 ± 65.7 mg/dL vs. 246.1 ± 97.9 mg/dL, p=0.166) blood glucose levels showed no significant differences between the two groups. We analyzed the C-peptide levels measured within one year of enrollment of this study. All enrolled patients were checked with fasting and postprandial C-peptide. The fasting C-peptide (2.38 ± 1.80 mg/dL vs. 2.29 ± 1.26 mg/dL, p=0.630) and postprandial C-peptide (3.83 ± 2.19 mg/dL vs. 4.14 ± 1.64 mg/dL, p=0.137) showed no significant differences between the two groups, but there was a significant difference in the total daily insulin doses (55.7 ± 23.6 U/day vs. 40.7 ± 20.8 U/day p< 0.05). There were no differences in baseline eGFR (82.74 ± 19.2 vs. 88.6 ± 20.9) and the prevalence of the hypertension (85% vs. 84%) or dyslipidemia (90% vs. 95%). There were no significant differences in the type of insulin or OHAs used in combination with insulin between the two groups. However, the frequency of basal insulin uses in group II seemed to be higher (16.1% vs. 26.8%, p=0.055).

**Table 1:** Clinical and laboratory characteristics of patients at baseline.

	Total	Group I	Group II	P-value
Patient Number	118	62	56	–
Age (yr)	60.4 ± 11.7	61.6 ± 9.7	59.1 ± 13.6	0.639
Sex (% male)	59%	63%	55%	0.407
BMI (kg/m2)	27.42 ± 3.50	27.60 ± 3.52	27.21 ± 3.50	0.590
Duration of Diabetes (yr)	14.2 ± 8.0	15.3v8.3	12.9 ± 7.5	0.103
Total Insulin Dose (Units)*	***48.5 ± 22.4***	***55.7 ± 23.6***	***40.7 ± 20.8***	***<0.05***
HbA1c (%)	8.62 ± 0.93	8.56 ± 0.78	8.69 ± 1.08	0.802
Fasting plasma glucose (mg/dL)	144.2 ± 51.9	140.8 ± 50.2	148.1 ± 53.8	0.351
Postprandial glucose (mg/dL)	229.4 ± 84.2	213.5 ± 65.7	246.1 ± 97.9	0.166
Fasting C-peptide (ng/mL)	2.34 ± 1.55	2.38 ± 1.80	2.29 ± 1.26	0.630
Postprandial C-peptide (ng/mL)	3.98 ± 1.94	3.83 ± 2.19	4.14 ± 1.64	0.137
eGFR (ml/min/1.73 m²)	85.5 ± 20.1	82.74 ± 19.2	88.6 ± 20.9	0.155
Hypertension (%)	85	85	84	0.815
Dyslipidemia (%)	92	90	95	0.379
Insulin				0.055
Basal insulin	25 (21.2%)	10 (16.1%)	15 (26.8%)	
Premixed insulin	89 (75.4%)	48 (77.4%)	41 (73.2%)	
MDI	4 (3.4%)	4 (6.5%)	0 (0%)	
OHAs with Insulin (%)				
DDP4i	53.3	50	57.1	0.136
Sulfonylurea	20	20.3	19.6	0.077
Metformin	70	73.4	66.1	0.398
SGLT2i	19.2	18.8	19.6	0.230
Etc.	5	3.1	7.1	0.542

BMI, body mass index; eGFR, Estimated glomerular filtration rate; MDI, Multiple daily injection; OHAs, oral hypoglycemic agents. Data are expressed as mean ± standard deviation. Sex (% male), OHAs with insulin (%), Hypertension (%), Dyslipidemia (%) analyzed by Chi-square test; other baseline characteristics analyzed by T-test. *P-value < 0.05, Group I vs Group II. Bold values for Statistically significant values (P-value < 0.05).

### Changes in Glycemic Parameters During the Follow-Up Period

**Figure 2:**
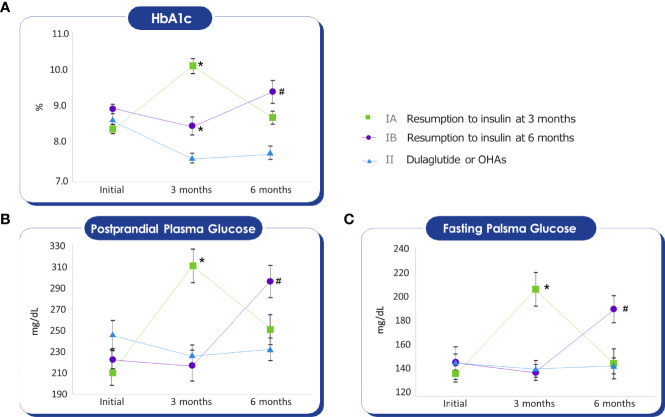
Mann-Whitney test was conducted to compare the average of the glycemic parameters with Group II. Initial HbA1c, Fasting glucose, Postprandial glucose values do not show differences between groups. **(A)** HbA1c of Group IA, IB at three months, and HbA1c of Group IB at six months were higher than Group II. *p-value < 0.05, ^#^p-value < 0.05. **(B)** Postprandial glucose of Group IA at three months, and Postprandial glucose of Group IB at six months were higher than Group II. *p-value < 0.05, ^#^p-value < 0.05. **(C)** Fasting glucose of Group IA at three months, and Fasting glucose of Group IB at six months were higher than Group II. *p-value < 0.05, ^#^p-value < 0.05.

The changes in the HbA1c levels during the follow-up period are shown in **Figure 2A**. In contrast to the HbA1c of group I, which increased at three months or six months, the HbA1c of group II was decreased and sustained after switching to weekly dulaglutide from insulin. In group Ia, the HbA1c level increased by 20% from baseline at 3 months (ΔHbA1c =1.7, p <0.05). In group Ib, the HbA1c level decreased by 4.5% (ΔHbA1c = -0.42) from baseline at 3 months, but increased by 5.6% (ΔHbA1c = 0.54) from baseline and 10.6% compared to 3 months (ΔHbA1c = 0.96, p<0.05), at 6 months. In group II, the HbA1c level decreased from 8.7% at baseline to 7.8% at 6 months after switching from insulin to dulaglutide (ΔHbA1c = -0.93, p <0.05). **Figures 2B, C**show the changes in fasting and postprandial blood glucose levels during the follow-up period, the trends in the change of postprandial and fasting plasma glucose levels during the follow-up period were similar to that of HbA1c.

**Figure 3:**
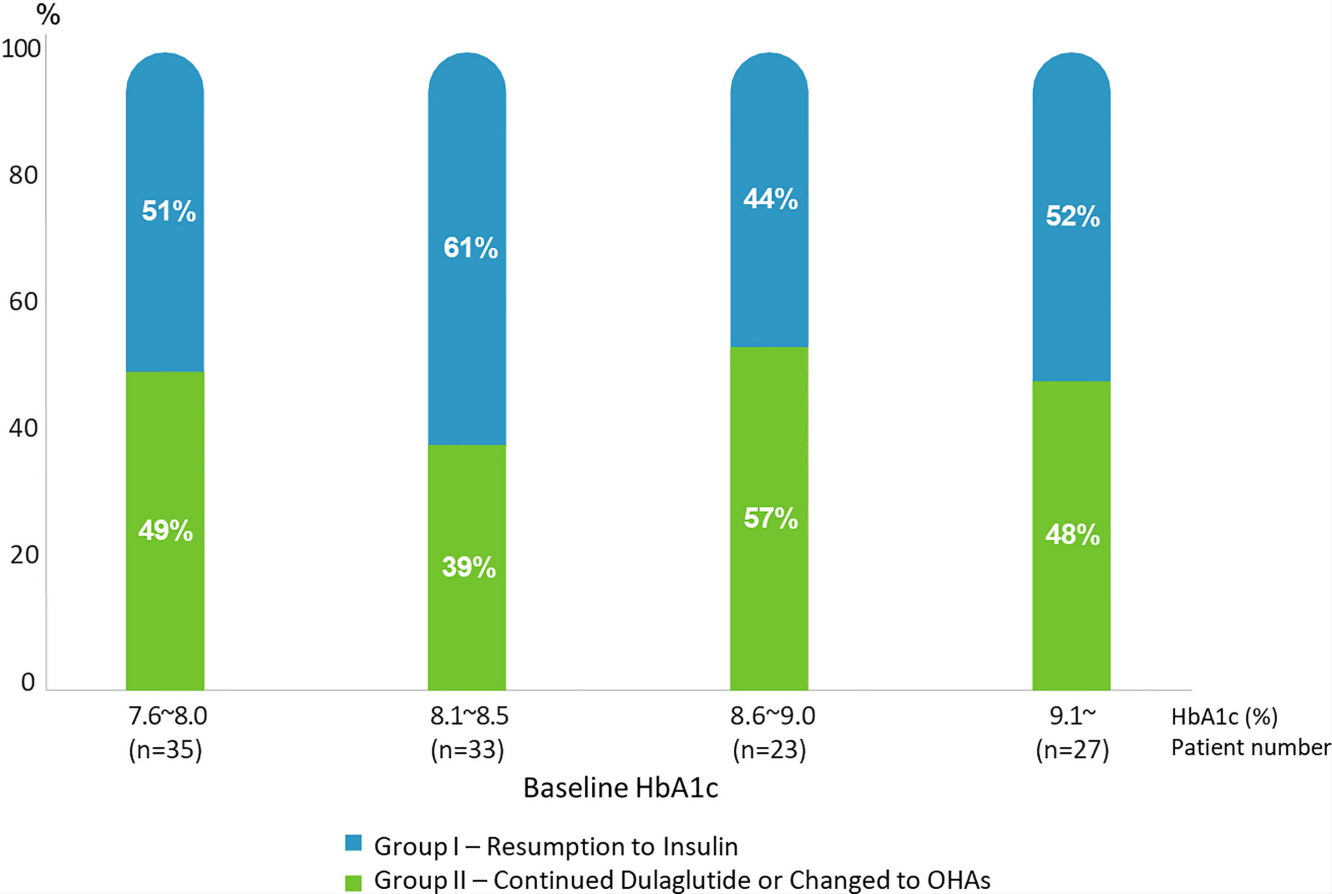
Rates of Resumption to Insulin (Group I) and Continued Dulaglutide/Changed to OHAs (Group II) according to baseline HbA1c range.

**Figure 3** demonstrates the change in the rate of insulin resumption during the follow-up period based on the baseline HbA1c categories. There was no significant relationship between insulin resumption and the baseline HbA1c levels (p=0.737). This shows that the possibility of insulin resumption is low in patients with relatively high postprandial blood glucose levels at baseline, which is consistent with the results of the logistic regression analysis described hereafter.

### Predictive Parameters for the Resumption of Insulin Therapy After Initial Insulin Discontinuation and Failure of Treatment With Dulaglutide

A logistic regression analysis was performed to identify the predictive parameters for the resumption of insulin therapy for glycemic control in patients who switched to dulaglutide from insulin therapy, and the results are shown in **Table 2**. We included clinically significant traditional factors and established parameters that were significantly different between groups I and II based on the results in **Table 1**. The results showed that younger age, a lower total daily dose of insulin, and higher postprandial plasma glucose levels were associated with lower risks of the resumption of insulin use after switching to dulaglutide from insulin as shown in **Table 2**.

**Table 2:** Logistic regression analysis of resumption to insulin after change from insulin to dulaglutide.

	Univariate				Multivariate			
	P-value	β^a^ (standardized)	Confidence interval		P-value	β^a^ (standardized)	Confidence interval	
Age	0.262	1.018	0.987	1.050	0.035*	***1.044***	***1.003***	***1.086***
Sex	0.405	0.731	0.350	1.528	0.446	–	–	–
BMI	0.547	1.033	0.930	1.146	0.710	–	–	–
Duration of Diabetes	0.115	1.309	0.991	1.090	0.569	–	–	–
Total Insulin Dose	***0.001†***	***1.032***	***1.013***	***1.052***	0.001*	***1.036***	***1.015***	***1.059***
HbA1c	0.457	0.861	0.581	1.277	0.532	–	–	–
Fasting plasma glucose	0.447	0.997	0.990	1.004	0.922	–	–	–
Postprandial glucose	***0.047†***	***0.995***	***0.990***	***1.000***	0.022*	***0.993***	***0.988***	***0.999***
Fasting C-peptide	0.773	1.036	0.816	1.314	0.911	–	–	–
Postprandial C-peptide	0.395	0.920	0.759	1.115	0.397	–	–	–
eGFR	0.114	0.985	0.967	1.004	0.420	–	–	–
Hypertension	0.815	1.128	0.413	3.007	0.943	–	–	–
Dyslipidemia	0.384	0.528	0.126	2.221	0.592	–	–	–
Type of Insulin	0.473	–	–	–	0.572	–	–	–

BMI, body mass index; eGFR, Estimated glomerular filtration rate † P-value < 0.05, Group I vs. Group II (Univariate logistic regression analysis), *P-value < 0.05, Group I vs. Group II (Multivariate logistic regression analysis). ^a^Results expressed as standardized β coefficient. Bold values for Statistically significant values (P-value < 0.05).

## Discussion

Individuals with T2D each have different pathogenic and clinical conditions in glucose metabolism (13). Interactions between genetic, environmental, and behavioral factors lead to considerable phenotypic variability, and this variability is reflected by heterogeneous responses to different drugs (8, 14). Considering both the limitations of insulin usage and the advantages of weekly GLP-1 receptor analog administration with respect to the ease of injection and therapeutic targeting toward the intestine, brain, and pancreas (15), we hypothesized the applicability of switching to weekly GLP-1 receptor analogs from insulin in patients with T2D whose HbA1c levels were 7.6% or higher. In this retrospective study of 138 subjects with T2D, there were two main findings. First, 20 patients with T2D (approximately 14.5%) could not tolerate or did not prefer weekly dulaglutide administrations (reasons included cost, gastrointestinal side effects, dissatisfaction with the drug), and 56 (approximately 40.6%) could successfully discontinue insulin and use either weekly dulaglutide or OHAs and demonstrated glycemic effectiveness after the switch. The mean HbA1c value in group II significantly reduced from 8.7% to 7.8%, and of the 56 group II patients, 23 (16.7%) patients could completely cease all injection therapies including dulaglutide and maintained stable glycemia over the 6-month period. Second, we found that older age, a higher dose of insulin at the time of switching to dulaglutide, and a low level of postprandial glucose were significant predictive factors for insulin resumption after switching from insulin to weekly dulaglutide.

Previous studies have investigated the effectiveness of switching from insulin to dulaglutide in reducing HbA1c levels and body weight in patients with T2D (16). In contrast to our study, the patients used a lower dose of insulin (about 20U/day), and approximately 94% of the enrolled patients used only basal insulin with OHAs. However, in our study, the average insulin dose was higher (mean total daily insulin dose, 48U/day), and approximately 78.8% of the patients were using premixed or basal–bolus insulin with prandial short-acting regimens. Moreover, the HbA1c level was also higher in our study compared to that in a previous study (8.6% vs. 8.2%). Regarding replacing preprandial short-acting insulin analogs with GLP-1 receptor agonists in poorly controlled glycemia despite intensive insulin regimens, the FLAT-SUGAR study indicated that basal insulin plus the mealtime administration of exenatide can be as effective in reducing HbA1c levels as basal–bolus insulin therapy (17). Kim et al. also reported that the dulaglutide and basal insulin combination therapy was as effective as basal–bolus insulin therapy in kidney transplant recipients with T2D (12). These studies indicated that basal insulin with GLP-1 receptor agonist therapy reduced the overall insulin dose and lowered body weight as compared to basal–bolus insulin therapy alone. This effect can be explained by the established glucose-dependent, glucose-lowering, and appetite-decreasing effects of GLP-1 receptor agonists that consequently result in marked reductions in postprandial glucose levels (15). However, few studies have investigated whether replacing insulin therapy with a combination of a GLP-1 receptor agonist and OHAs could be effective in patients with uncontrolled T2D receiving insulin therapy.

With respect to tolerability and the effectiveness of switching to weekly dulaglutide from insulin therapy, approximately 40.6% of the patients did not resume insulin treatment in this study. This unexpected high success rate of switching from insulin treatment to weekly GLP-1 receptor agonist therapy might be explained by the GLP-mediated improvement in insulin resistance and secretion, and its strong beneficial effect in controlling glycemic excursion (15). Low postprandial glucose levels at the time of switching were associated with a higher risk of insulin resumption after switching to dulaglutide. This can support the effectiveness of GLP-1 receptor agonists on postprandial glucose levels. Moreover, the frequency of injections dramatically reduced from more than one insulin injection per day to a weekly GLP-1 receptor agonist; therefore, this might lead to satisfaction and preference for the GLP-1 receptor agonist treatment in patients, and it may consequently enhance treatment compliance. Despite the effectiveness of insulin in controlling hyperglycemia, it has limitations as patients and physicians are reluctant to intensify insulin treatment due to side effects such as hypoglycemia, weight gain, and the inconvenience of frequent injections in clinical practice.

The predictive clinical factors for the resumption of insulin therapy to maintain the optimal glucose control in Korean patients with T2D were as follows: 1) total daily insulin dose at baseline, regarding daily insulin doses, we noted that using the receiver-operating characteristic analysis with a total daily insulin dose of >44U was the cutoff value for predicting insulin resumption, 2) postprandial glucose level, and 3) older age. It is expected that the baseline HbA1c and C-peptide levels may be the key predictive factors to resume insulin therapy after failure of change from insulin to dulaglutide or continue changing to dulaglutide. Interestingly, neither the initial HbA1c levels (when patients shifted from insulin to dulaglutide) nor C-peptide levels were independent factors predicting the resumption of insulin therapy in this study. Even in individuals with HbA1c levels > 9%, 48% of the patients were able to continue GLP-1 receptor agonist treatment and did not resume insulin treatment. We postulated that some enrolled patients may be poorly compliant with the insulin injections and lifestyle modification. Additionally, high insulin dosage and multi-daily insulin injections may contribute to poor compliance. Although information on the frequency or severity of hypoglycemia after treatment change is lacking, Dulaglutide may improve compliance by reducing unfavorable hypoglycemia, compared to insulin. Moreover, dulaglutide’s beneficial effects on satiety factored into its effects in glycemic control independent from the baseline parameters. Finally, most patients that were included in this study still had sufficient insulin secretion and average HbA1c was approximately 8.5%. In this study, those who were successfully shifted to weekly dulaglutide had improved glycemic control. Based on the postulations, the baseline HbA1c and C-peptide levels were not the predictive factors in resuming insulin therapy after failure of changing from insulin to dulaglutide or continuing the switch to dulaglutidein this study. The finding suggests that the switch from insulin therapy to GLP-1 receptor agonist therapy can be done in patients with T2D who are younger and have a relatively low dose of insulin and with relatively high postprandial glucose levels even if they have higher HbA1c levels upon changing to dulaglutide. However, we have come up with a few reasons concerning that they did not have great influence in our study.

The present study has several limitations that should be addressed in further studies. First, this study was designed as an uncontrolled, open-label, longitudinal, retrospective study, which is limited in its applicability and clinical relevance to generalization and broader clinical practice. However, our findings show the natural results of real-world practice that did not involve any interventions. Second, as the proposed switch therapy was not a guideline-based accepted approach, a relatively small number of patients were enrolled in this study. Furthermore, the short follow-up period is a limitation of our study, potentially limiting the generalization of our results. Moreover, the change in anti‐diabetes medications before and after initiating dulaglutide treatment may also influence the final results. Third, we could not collect data on patient satisfaction, compliance or adverse events including hypoglycemia with dulaglutide use,. Nevertheless, the present study clearly demonstrated that the proposed switch method may benefit a significant number of patients even when hyperglycemia is uncontrolled with high doses and multiple injections of insulin.

In conclusion, dulaglutide, a weekly GLP-1 receptor agonist, can be used for glycemic control in patients with T2D with glucose levels inadequately controlled by insulin regimens. The tolerability and effectiveness of dulaglutide were not dependent on HbA1c level at the time of switching to dulaglutide from insulin. Older age, a relatively high daily insulin dose (> 44U/day), and a lower level of postprandial glucose at baseline were clinical predictive characteristics for the resumption of insulin after switching to dulaglutide. Large-scale, long-term, randomized controlled studies are needed to generalize our findings and to accurately analyze the efficacy of the present approach.

## Data Availability Statement

The raw data supporting the conclusions of this article will be made available by the authors, without undue reservation.

## Author Contributions

YK and JH contributed equally to this work. YK, study design, data acquisition, data analysis and interpretation, and manuscript writing. JH and B-WL, study design, data analysis and interpretation, and manuscript writing. ML, EK, and B-SC, data analysis and interpretation. All authors have read and approved the final manuscript.

## Conflict of Interest

The authors declare that the research was conducted in the absence of any commercial or financial relationships that could be construed as a potential conflict of interest.

## Publisher’s Note

All claims expressed in this article are solely those of the authors and do not necessarily represent those of their affiliated organizations, or those of the publisher, the editors and the reviewers. Any product that may be evaluated in this article, or claim that may be made by its manufacturer, is not guaranteed or endorsed by the publisher.
